# Carotid endarterectomy in patients with recurrent symptoms associated with an ipsilateral carotid artery near occlusion with full collapse

**DOI:** 10.1007/s00415-018-8939-z

**Published:** 2018-06-18

**Authors:** A. J. A. Meershoek, E. P. A. Vonken, P. J. Nederkoorn, L. J. Kappelle, G. J. de Borst

**Affiliations:** 1 Department of Vascular Surgery, Room G04.129, University Medical Centre Utrecht, Utrecht University, PO Box 85500, 3508 GA Utrecht, The Netherlands; 2 Department of Radiology, University Medical Centre Utrecht, Utrecht University, Utrecht, The Netherlands; 3 Department of Neurology, Academic Medical Centre Amsterdam, Amsterdam, The Netherlands; 4 Department of Neurology, University Medical Centre Utrecht, Utrecht University, Utrecht, The Netherlands

**Keywords:** Carotid artery stenosis, Carotid endarterectomy, Near occlusion, Best medical treatment

## Abstract

**Objective:**

Near occlusion (NO) of the internal carotid artery (ICA) with full collapse (NOFC) is a rare condition, with a prevalence of around 1%. Guidelines on carotid stenosis recommend a conservative treatment in patients with a single-event ipsilateral to a NOFC, but the optimal treatment for patients with recurrent symptoms associated with NOFC remains uncertain. We describe a consecutive series of patients with recurrent symptoms associated with NOFC (RSNOFC) who underwent carotid endarterectomy (CEA).

**Methods:**

From 2008 to 2017, 17 consecutive patients with RSNOFC were treated according to our standardized multidisciplinary work-up and protocol and included for this single-center cohort study. NO was defined according to the angiographic North American Symptomatic Carotid Endarterectomy Trial criteria. Only patients with NOFC were included in this study.

**Results:**

Standard longitudinal CEA was performed in 15 patients, whereas in 2 patients the ICA was ligated with concomitant endarterectomy of the ECA. Within 30 postoperative days, one patient died from a hemorrhagic infarction. During follow-up (median 23 months) one patient died of unknown cause 90 days after CEA. No TIA, stroke, myocardial infarction or re-stenosis occurred in the remaining patients.

**Conclusion:**

In patients with RSNOFC, CEA may be considered a potential treatment option. Although procedural risks in this small subgroup may be higher as compared to patients with low-to-moderate risk anatomy, this risk may outbalance the natural course.

## Introduction

The term internal carotid artery near occlusion (ICA NO) is used for a severe stenosis of the internal carotid artery with a collapse of the artery distal of the stenosis [1]. It is a relatively rare condition, with a prevalence of around 20% amongst all patients with a severe (> 70%) symptomatic carotid artery stenosis and only 1% of these patients have an ICA NO with full collapse [2]. The first relevant angiographic criteria to diagnose ICA NO were defined in 1997 by the North American Symptomatic Carotid Endarterectomy Trial (NASCET) [3] and specified in 2005 [2]. Nevertheless, published cohort studies have used different and heterogeneous definitions of ICA NO during the past years (for example near total occlusion, pseudo-occlusion, string sign) [4–8]. As a result, for the domain of patients with carotid artery stenosis, no consensus on the definition of near occlusion has yet been reached.

Optimal medical treatment (OMT) has been recommended for patients with an ICA NO [9], which is based on a reanalysis of the European Carotid Surgery Trial (ECST-1), NASCET and the Veterans Affairs trial 309 [10]. This pooled analysis revealed that in patients with ICA NO (irrespective of the presence or absence of a post-stenotic full collapse), CEA plus OMT had a negative absolute risk reduction in 5 year follow-up (absolute risk reduction − 0.1%) [10]. From this time onwards, OMT was recommended in patients with symptomatic ICA NO and these patients were excluded from randomized controlled trials. Since then, a wide variety in cohort studies was published on treatment approaches and results [4–8, 11–20]. More recently, a meta-analysis (mainly based on small cohort studies) could not provide a new, definitive recommendation for clinical practice because of the limited number of included studies and patients, the use of a variety in diagnostic criteria, and the lack of a distinction between ICA NO with or without a post-stenotic full collapse [21].

The distinction between ICA NO with or without a post-stenotic full collapse may be of clinical relevance since natural course cohort studies described 12.5 and 43% of recurrent events within 90 days [8, 19]. In addition, in the NASCET study, the 1-year risk of ipsilateral stroke in patients with a NO with full collapse (NOFC) was 6.7% when treated surgically and 11.1% when treated medically [3]. No statements were made regarding the distinction between symptomatology and recurrent symptomatology.

For clinicians treating patients with cerebrovascular disease, the subpopulation of patients with recurrent symptomatic NOFC (RSNOFC) poses a serious clinical challenge. For this subgroup no clear guideline recommendations exist and the optimal treatment approach is uncertain [22].

Due to the lack of available evidence in the literature with regards to the best treatment approach for this high-risk subgroup of patients with RSNOFC, we assessed the treatment results of a selected series of patients in our tertiary vascular referral center.

## Materials and methods

Ethical approval was obtained from the local Research Ethics Committee, UMC Utrecht the Netherlands. No formal informed consent was needed from the participants in this study.

### Patient selection

Consecutive patients who underwent CEA for high-grade symptomatic carotid artery stenosis in a single-center university hospital in the Netherlands between January 2008 and June 2017 were retrospectively screened for the presence of NOFC. Eligible patients were collected out of multi-disciplinary rounds. Patient information was prospectively collected in a dedicated database. Patients with a symptomatic NOFC were initially treated (or optimized) with adequate medical therapy. Patients with recurrent symptoms associated with NOFC, which we defined as recurrent symptoms despite OMT, underwent CEA after multidisciplinary discussions between vascular surgeons, neuro-radiologists and neurologists. This is our standard multidisciplinary treatment approach. All patients received extensive information about this out of protocol therapy and agreed to the procedure. The patients with RSNOFC despite OMT who underwent CEA were included in this study.

The following baseline characteristics were collected: (1) demographics (age, gender), (2) clinical characteristics (medical history, type of neurological symptoms), and (3) use of medication, (4) imaging characteristics.

### Definition of ICA NO with a post-stenotic full collapse

Standard preoperative imaging was performed in all patients. All patients underwent a carotid duplex in combination with either a magnetic resonance angiography (MRA) or computerized tomography angiography (CTA) of the carotid arteries. Only in cases with discrepancy between the two applied imaging modalities, an additional diagnostic substraction angiography (DSA) was performed.

**Fig. 1:**
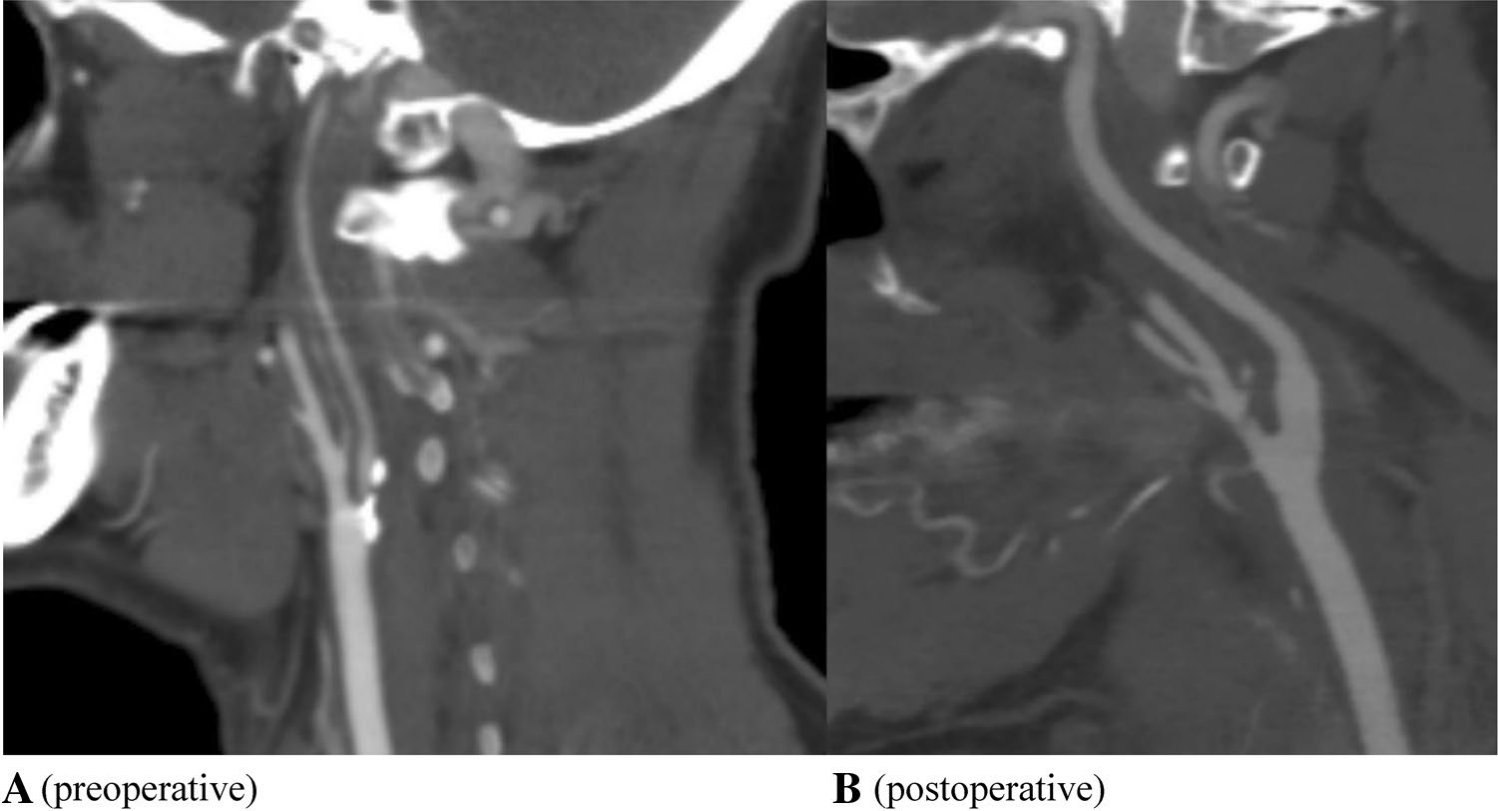
The preoperative and postoperative internal carotid artery. In **a** the internal carotid artery is almost full collapsed, an internal carotid artery near occlusion with a post-stenotic full collapse. In **b**, the internal carotid artery is reconstituted to a normal diameter after carotid endarterectomy

In 2017, all patients were retrospectively evaluated according to the angiographic criteria for ICA NO from the NASCET, whereby recognition of two or more of the criteria was used to categorize the prominent stenosis as an ICA NO [2, 3]. These patients were further subdivided visually by the presence or absence of a post-stenotic (tiny thread-like) lumen according to the NASCET criteria (see Fig. 1) [2, 3]. Only patients with an ICA NO with a post-stenotic tiny thread-like lumen, which is called a post-stenotic full collapse, were included in this study. In our study, patients with a post-stenotic lumen of less than 2.5 mm were identified as full collapse.

### Optimal medical therapy

All patients were initially treated by optimal medical therapy, including standard antiplatelet therapy and lipid lowering medication, and if necessary antihypertensive or antidiabetic medication [23]. Standard antiplatelet therapy includes monotherapy with clopidogrel or dual therapy with acetylsalicylic acid and dipyridamole [9].

### Surgical procedure

Conventional longitudinal CEA technique was performed under general anesthesia with neuro-monitoring by four different vascular surgeons. After exposure of the carotid bifurcation, the common carotid artery and the ICA were first clamped on trial, to decide if a shunt was needed. A shunt was used in case of electroencephalographic asymmetry. Heparin was administered intravenously. A longitudinal arteriotomy was made on the ICA with subsequent removal of the atherosclerotic plaque. Verification of the presence of backflow of the ICA was done and if necessary, the ICA was stretched with a coronary probe to a maximum of 2.5 mm for adequate patch angioplasty. If no backflow of the ICA was noted, the ICA was ligated while the external carotid artery flow was preserved. Closure was performed with a patch (bovine) angioplasty.

All patients were monitored continuously during surgery using electroencephalography (EEG) and transcranial Doppler (TCD). Standard postoperative care was performed according to the hospital protocol, which includes neuro-monitoring by TCD 2 and 24 h after carotid revascularization [24]. All patients with postoperative hypertension, defined as (1) systolic blood pressure (SBP) > 160 mmHg, (2) SBP increase > 20% compared to the preoperative SBP, or (3) increase of > 100% of the intraoperative mean flow velocity of the ipsilateral middle cerebral artery, underwent strict blood pressure control. If the blood pressure was not sufficiently controlled, irrespective of neurological symptomatology, the patient was transferred to the medium care unit.

### Outcome

The primary outcome was defined as the occurrence of any stroke, death, myocardial infarction or transient ischemic attack (TIA) within the first 30 days of treatment. Secondary outcomes were: (1) occurrence of stroke, death, myocardial infarction or TIA beyond 30 days of treatment and (2) occurrence of re-stenosis, other complications (wound infection, dysphonia and non-fatal hyperperfusion syndrome).

Re-stenosis was defined as presence of more than 50% stenosis in the operated ICA according to the NASCET criteria for carotid stenosis, as diagnosed on CTA, MRA or duplex ultrasound (Peak Systolic Velocity of more than 125 cm/s).

Follow-up included clinical assessment at the outpatient clinic and a duplex ultrasound (or CTA or MRA) of the carotid arteries 6 weeks to 3 months after the operation. Afterwards patients were seen at the outpatient clinic on a yearly basis.

### Statistical analysis

Data were entered and analyzed in SPSS (IBM SPSS Statistics for Windows, Version 23.0. Released 2015. Armonk, NY: IBM Corp.). Categorical variables were presented as frequencies. Continuous variables were presented as medians with interquartile ranges (IQR). No formal statistical analysis could be conducted because of the limited sample size.

## Results

### RSNOFC criteria

In total, 913 CEAs were performed during the study period. 20 patients were identified with symptomatic NOFC, of which 17 patients had recurrent symptoms despite protocolised and monitored OMT.

A median peak systolic velocity of 15 cm/s (range 0–60) was found in these 17 patients on duplex in the extracranial distal internal carotid artery.

### Baseline characteristics

The baseline characteristics are shown in Table 1. The median age was 65 years (IQR 44–81 years). 14 patients presented with recurrent TIAs and 3 patients had recurrent non-disabling ipsilateral ischemic strokes (all within a period of 8 months). All patients were treated with antiplatelet agents (Table 1). One patient received acetylsalicylic acid monotherapy because of a gastric bleeding during dual antiplatelet therapy (acetylsalicylic acid and dipyridamole) in the past.

**Table 1:** Baseline characteristics

Characteristics	Total 17 patients
Male:female (*n*)	12:5
Age (years)	65 (IQR 44–81)
Diabetes mellitus (*n*)	4
Hypercholesterolemia (*n*)	13
Contralateral stenosis > 50% (*n*)	0
Symptomatic (*n*)	
Recurrent TIAs	14
Recurrent ipsilateral ischemic stroke	3
Antiplatelet medication (*n*)	
Acetylsalicylic acid + dipyridamole	10
Acetylsalicylic acid + clopidogrel	1
Clopidogrel	5
Acetylsalicylic acid	1

*n* numbers/the amount of patients, *IQR* interquartile range, *TIA* transient ischemic attack

### Treatment

A conventional longitudinal CEA was performed in 15 patients while in 2 patients the ICA was ligated with concomitant external carotid artery endarterectomy. None of the 17 patients were shunt-dependent during cross-clamping during intraoperative monitoring.

### Clinical outcome and follow-up

One patient in whom a CEA was performed, suffered from a hemorrhagic infarction 5 days after the procedure due to a hyperperfusion syndrome and died afterwards (Table 2). This patient developed postoperative hypertension and received strict blood pressure control with an intravenous beta blocker (labetolol). No other strokes, myocardial infarction or TIAs were reported in the remaining patients within 30 days.

**Table 2:** Outcome

Outcome	Total patients (numbers)
Outcome < 30 days (*n*)	
TIA	0
Stroke	0
Myocardial infarction	0
Death	1
Outcome > 30 days (*n*)	
TIA	0
Stroke	0
Myocardial infarction	0
Death	1
Other complications (wound infection, dysphonia, and non-fatal hyperperfusion syndrome)	0
Re-stenosis > 50% (*n*)	0
Median follow-up period (months)	23 (range 2–71)

*n* numbers/the amount of patients, *TIA* transient ischemic attack

Clinical follow-up was performed in all patients. The median follow-up period was 23 months (range 2–71 months). No strokes, myocardial infarctions or re-stenosis were seen beyond 30 days after the procedure. One patient had died of unknown cause 90 days after CEA. 6 weeks after CEA no complications were reported at the outpatient clinic in this patient and the patient had a patent internal carotid artery on CTA.

In the revascularized patients, the ICA diameters were equal to the contralateral non-stenosed carotid artery on postoperative imaging with CTA (Fig. 1).

## Discussion

In 17 patients with RSNOFC despite adequate medical treatment who underwent CEA, we show a 30-day post-procedural stroke/death rate of 5.9%. Besides this, no re-stenosis was seen on CTA, MRA or duplex ultrasound during the follow-up period.

Our relatively low peri- and post-procedural stroke/death rate is similar to the stroke risk in surgically treated patients with an ICA NO participating in the ECST-1 and NASCET, (irrespective of presence or absence of a post-stenotic full collapse) [10]. The negative absolute risk reduction after 5 year follow-up in patients treated with CEA in the pooled analysis is remarkable [10], as a beneficial effect was suggested by the NASCET investigators since in patients with NOFC, the 1-year ipsilateral stroke risk was 11.1% in medically treated patients versus 6.7% in surgically treated patients [3]. An explanation for this could be that the NASCET reported the 1-year ipsilateral stroke risk while the pooled analysis reported the combined any stroke and operative death risk. Besides this, ECST patients had a lower prevalence of vascular risk factors such as advanced age, ischemic heart disease and dyslipidemia [2].

Since patients with ICA NO (with and without a post-stenotic full collapse) were identified in the first landmark trials as a subgroup that did not benefit from CEA, these patients were excluded from subsequent RCTs. As a consequence, only outdated data and data from cohort studies are available about the pros and cons of CEA, CAS or BMT in these patients.

A limited number of cohort studies have published the results of their centers’ preferred treatment approach, which varied from CEA [11–13] to carotid artery stenting (CAS) [4–7, 14–18] or BMT [8, 12, 19]. Reported outcomes were highly heterogeneous and ranged from a 30-day stroke/death risk of 0–11.1% for CEA [11–13, 20] to 0–9.2% for CAS [5–7, 16–18] and 0–43% for best medical treatment (BMT) [8, 12, 19]. Furthermore, analyses of published studies are hampered by both a variety in inclusion criteria and the reporting of different clinical outcomes. In addition, most of the cohort studies do not distinguish between patients with an ICA NO with or without a post-stenotic full collapse, nor between symptomatic and asymptomatic patients, even though evidence suggests this distinction to be highly important [8]. Only the NASCET distinguished between ICA NO with versus without a post-stenotic full collapse and reported a 30-day procedural risk of 6.7% in patients with a NOFC which was similar to this risk in patients with a 70–94% [3]. Since the 30-day procedural CEA risk decreased over the last years [25], it is unfortunate that no recent data are available on this risk in patients with NOFC in a randomized setting.

The procedural risks may still be higher in patients with NOFC than in patient with regular risk anatomy, mainly because of technical reasons and due to hemodynamic issues such as hyperperfusion. Therefore, part of this deemed higher stroke risk in the peri-procedural phase may be preventable by adequate measures for hemodynamic control [13, 20, 26]. Information about the natural course in patients with NOFC has been published only in one national multicenter study and in one single-center cohort study [8, 19]. In, respectively, 12.5 and 43% of the patients with NOFC, a recurrent event within 90 days has been observed. In the current study, a high percentage of patients had recurrent events despite adequate medical therapy (85%, 17 out of 20) and of them 15% had recurrent strokes. Our patients were seen on a yearly basis at the outpatient clinic after their first event. This could lead to a higher detection rate of recurrences. We observed recurrent events ranged from within 1 month to several years within these patients.

The reason why patients with NOFC do suffer from recurrent symptoms is still unclear. Several pathophysiological mechanisms have been described [27]. This is the first study to report data using EEG-guided artery clamping in these patients with NOFC. In our case series, shunting was not necessary; which might implicate that a thrombo-embolic problem is the underlying cause, rather than a hemodynamic component. We have found that these cases actually have normal ICA diameters after CEA as the ICA diameter is small simply due to low pressure, and reconstitute to normal diameter when pressurized (Fig. 1). Therefore, in our opinion, optimal medical treatment with adequate antiplatelet therapy could be considered an important role in the treatment of this high-risk patient group.

The strength of the current is study is its consecutive design with standard decision-making process including the expertise of vascular surgeons, neuro-radiologists and neurologists. Our study reported results with a relatively long follow-up period compared to other cohort studies. A limitation is the retrospective study design. Since we included consecutive patients who were treated according to a strict protocol, the retrospective nature probably has little influence on our results. Because of the small sample size, we could not perform solid statistical analyses. In addition, no parallel cohort was available which described solely the conservative treatment of these patients.

In conclusion, in patients with RSNOFC, CEA may be considered a potential treatment option. Although procedural risks in this small subgroup may be higher as compared to patients with low-to-moderate risk anatomy, this risk may outbalance the natural course.
