# Correction to: Carotid endarterectomy in patients with recurrent symptoms associated with an ipsilateral carotid artery near occlusion with full collapse

**DOI:** 10.1007/s00415-018-9021-6

**Published:** 2018-08-27

**Authors:** A. J. A. Meershoek, E. P. A. Vonken, P. J. Nederkoorn, L. J. Kappelle, G. J. de Borst

**Affiliations:** 1 Department of Vascular Surgery, Room G04.129, University Medical Centre Utrecht, Utrecht University, PO Box 85500, 3508 GA Utrecht, The Netherlands; 2 Department of Radiology, University Medical Centre Utrecht, Utrecht University, Utrecht, The Netherlands; 3 Department of Neurology, Academic Medical Centre Amsterdam, Amsterdam, The Netherlands; 4 Department of Neurology, University Medical Centre Utrecht, Utrecht University, Utrecht, The Netherlands

## Correction to: Journal of Neurology (2018) 265:1900–1905 10.1007/s00415-018-8939-z

The original version of this article unfortunately contained a mistake. The copyright line was incorrect in the HTML version. The correct copyright line should be “The Author(s) 2018”.

